# The Effect of Pore Size Distribution and l-Lysine Modified Apatite Whiskers (HAP) on Osteoblasts Response in PLLA/HAP Foam Scaffolds Obtained in the Thermally Induced Phase Separation Process

**DOI:** 10.3390/ijms22073607

**Published:** 2021-03-30

**Authors:** Konrad Szustakiewicz, Marcin Włodarczyk, Małgorzata Gazińska, Karolina Rudnicka, Przemysław Płociński, Patrycja Szymczyk-Ziółkowska, Grzegorz Ziółkowski, Monika Biernat, Katarzyna Sieja, Michał Grzymajło, Piotr Jóźwiak, Sylwia Michlewska, Andrzej W. Trochimczuk

**Affiliations:** 1 Department of Polymer Engineering and Technology, Faculty of Chemistry, Wrocław University of Science and Technology (WUST), Wyb. Wyspiańskiego 27, 50-370 Wrocław, Poland; malgorzata.gazinska@pwr.edu.pl (M.G.); katarzyna.sieja@pwr.edu.pl (K.S.); michal.grzymajlo@pwr.edu.pl (M.G.); andrzej.trochimczuk@pwr.edu.pl (A.W.T.); 2 Department of Immunology and Infectious Biology, Faculty of Biology and Environmental Protection, University of Łódź, Banacha 12/16, 90-237 Łódź, Poland; marcin.wlodarczyk@biol.uni.lodz.pl (M.W.); karoli-na.rudnicka@biol.uni.lodz.pl (K.R.); przemyslaw.plocinski@biol.uni.lodz.pl (P.P.); 3 Centre for Advanced Manufacturing Technologies, Faculty of Mechanical Engineering, Wrocław University of Science and Technology (WUST), Łukasiewicza 5, 50-370 Wrocław, Poland; patrycja.e.szymczyk@pwr.edu.pl (P.S.-Z.); grzegorz.ziolkowski@pwr.edu.pl (G.Z.); 4 Department of Biomaterials, Ceramic and Concrete Division, Łukasiewicz Research Network Institute of Ceramics and Building Materials, 02-676 Warsaw, Poland; m.biernat@icimb.pl; 5 Department of Invertebrate Zoology and Hydrobiology, Faculty of Biology and Environmental Protection, University of Łódź, Banacha 12/16, 90-237 Łódź, Poland; piotr.jozwiak@biol.uni.lodz.pl; 6 Laboratory of Microscopic Imaging and Specialized Biological Techniques, Faculty of Biology and Environmental Protection, University of Łódź, Banacha 12/16, 90-237 Łódź, Poland; sylwia.michlewska@biol.uni.lodz.pl

**Keywords:** polymers, foam scaffolds, PLLA, hydroxyapatite, cell adhesion, TIPS

## Abstract

In this research, we prepared foam scaffolds based on poly(l-lactide) (PLLA) and apatite whiskers (HAP) using thermally induced phase separation technique supported by the salt leaching process (TIPS-SL). Using sodium chloride having a size of (a) 150–315 μm, (b) 315–400 μm, and (c) 500–600 μm, three types of foams with different pore sizes have been obtained. Internal structure of the obtained materials has been investigated using SEM as well as μCT. The materials have been studied by means of porosity, density, and compression tests. As the most promising, the composite prepared with salt size of 500–600 μm was prepared also with the l-lysine modified apatite. The osteoblast hFOB 1.19 cell response for the scaffolds was also investigated by means of cell viability, proliferation, adhesion/penetration, and biomineralization. Direct contact cytotoxicity assay showed the cytocompatibility of the scaffolds. All types of foam scaffolds containing HAP whiskers, regardless the pore size or l-lysine modification induced significant stimulatory effect on the cal-cium deposits formation in osteoblasts. The PLLA/HAP scaffolds modified with l-lysine stimulated hFOB 1.19 osteoblasts proliferation. Compared to the scaffolds with smaller pores (150–315 µm and 315–400 µm), the PLLA/HAP foams with large pores (500–600 µm) promoted more effective ad-hesion of osteoblasts to the surface of the biomaterial.

## 1. Introduction

One of the most interesting and prospective materials used in bone tissue engineering is a composite based on poly(l-lactide) (PLLA) doped with apatite ceramics [1,2,3,4,5,6]. The composite consists of biodegradable and biocompatible PLLA which has also acceptable mechanical properties and can be easily processed using e.g., 3D printing techniques [7]. However, PLLA degradation causes formation of lactic acid which may lead to acidification of the environment and eventually inflammation in tissue [8]. The polymer is also hydrophobic [9]. These properties limit the application of PLLA in bone tissue engineering. PLLA doping with phosphate ceramics, i.e., hydroxyapatite, partially compensates the disadvantages of PLLA. Hydroxyapatite HAP is the main inorganic component of mammals’ bone tissue (~58%) but can also be artificially synthesized [10]. As a bioactive and osteoconductive material, HAP has been widely used as an implant for bone regeneration [5,10,11,12,13]. Among synthetic HAPs, micro-sized apatite whiskers are described in the literature to improve bioactivity bone binding as well as good mechanical properties [14].

Bioresorbable polymer composites can be prepared in several ways, including melt mixing–extrusion [13,15] and solvent techniques: solution casting [16] electrospinning [17,18], and thermally induced phase separation (TIPS) [18,19,20]. The last of these techniques enables one to prepare composites in the form of a foam, which can be used as the so-called scaffold that stimulates bone cells to grow on its surface [21,22]. The porosity of scaffolds obtained using TIPS is usually over 90% [23,24], which was found to increase the proliferation rate [25]. Cell migration and penetration is also correlated with pore architecture, i.e., pore size distribution and pore interconnectivity and surface area [26].

Amino acids such as l-arganine and l-lysine are essential compounds involved in bone metabolism and growth [27,28]. There are several studies concerning l-lysine modification of hydroxyapatite. It was found that surface modification of hydroxyapatite with l-lysine improves the dispersion and enhances the mechanical properties as well as in vitro bioactivity of polymeric scaffolds [29]. l-lysine promotes cell growth and osteogenic differentiation [30].

In this paper, PLLA with 50 wt.% of hydroxyapatite whiskers hybrids were obtained using the thermally induced phase separation technique. Three types of foam scaffolds were obtained using sodium chloride as porogen for different pore size distributions (150–315 µm, 315–400 µm, and 500–600 µm). As the most promising material, PLLA/HAP with 500–600 µm pores was also prepared using the l-lysine modified HAP. The effects of sodium chloride grain size on the pore size distribution, porosity, and compressive strength as well as osteoblasts response was investigated in the PLLA/HAP composite scaffolds.

## 2. Results and Discussion

### 2.1. Apatite Properties

**Figure 1:**
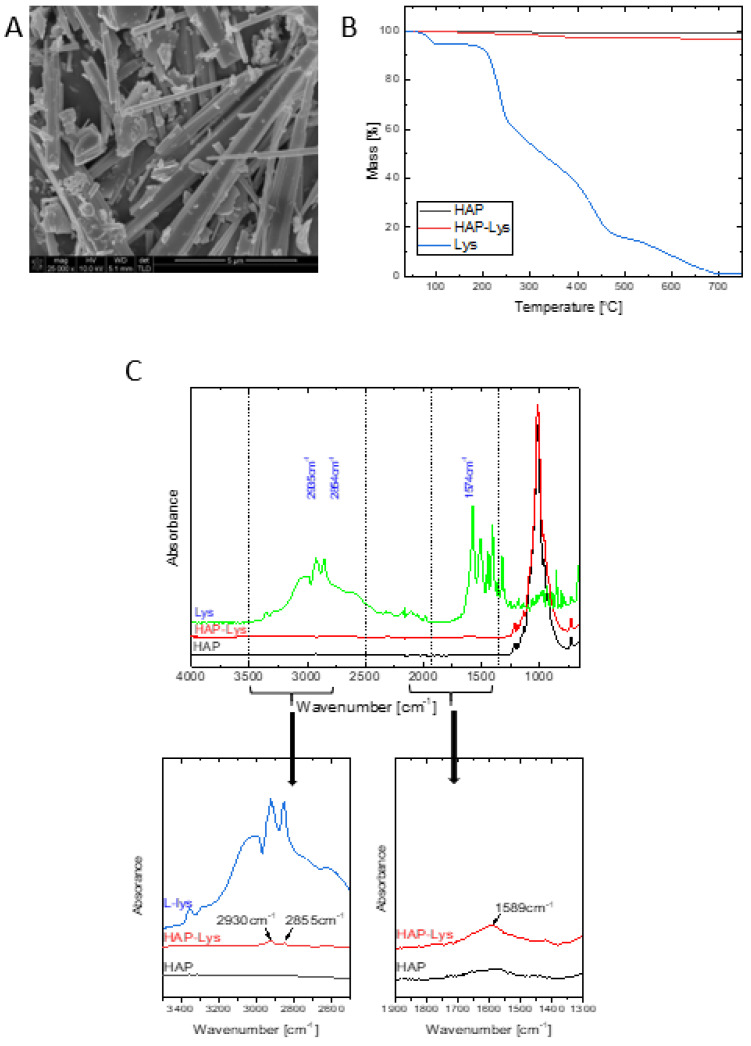
Characteristics of the obtained and modified hydroxyapatite (HAP): (**A**) SEM image of HAP whiskers, (**B**) TGA of l-lysine, HAP and HAP modified with l-lysine, (**C**) FTIR-ATR spectra of l-lysine, HAP and HAP modified with l-lysine.

The HAP whiskers obtained by the above mentioned one-pot synthesis were characterized earlier and revealed a triphasic product containing hydroxyapatite, whitlockite and calcium pyrophosphate in the amount of approximately: 72%, 15%, and 12%, respectively [31]. It was also shown that the high content of hydroxyapatite in the composition of whiskers can provide bioactivity and osteoconductivity in the final materials [32]. On the other hand, the presence of the whitlockite and calcium pyrophosphate phases may play an important role in biodegradable composites as their degradation rate is greater than that of neat hydroxyapatite [33,34]. The mean length of the whiskers was 18.84 ± 4.06 μm and their mean width was 1.13 ± 0.24 μm. A SEM image of the obtained whiskers is shown in Figure 1A. 

The l-lysine content on the HAP surface was determined from the TGA curves presented in Figure 1B. The l-lysine amount was calculated from the difference in mass loss at 700 °C of unmodified and surface modified HAP, with consideration of 98.66% of mass loss of neat l-lysine. The mass loss at 700 °C of unmodified HAP and HAP modified with l-Lysine was 0.94% and 3.12%, respectively. The l-lysine content on the surface of HAP reached 2.23 *wt*%; less than 10% of the amount of l-lysine taken in the modification procedure has been attached. These results agree with our previous results of the modification of nanohydroxyapatite particles [16]. 

The functionalization of HAP surface with l-lysine was conducted based on known interaction mechanism. On the surface of HAP particles Ca^2+^-rich region are located. The Ca^2+^-site of HAP binds to the negatively charged anionic groups such as carboxyl COO^−^, providing the ability of incorporation of biological molecules such as amino acids to the surface of HAP [35]. Qualitative and quantitative analyses of l-lysine molecules incorporated on the HAP surface and the interaction mechanism were described by Kollath et al. [36].

The chemical nature of the surface modified HAP whiskers was investigated using FTIR spectroscopy. The FTIR-ATR spectrum of pure l-lysine combined with FTIR-ATR spectra of HAP and HAP-Lys is shown in Figure 1C.

In the spectrum of HAP-Lys there are characteristic bands of hydroxyapatite, whitlockite, calcium pyrophosphate [31] and l-lysine. The bands at 2930 cm^−1^ and 2855 cm^−1^ from the alkane chain of l-lysine and the characteristic amide II band at 1589 cm^−1^ confirm the presence of an amino acid on the surface of HAP. Moreover, the bands at 1589, 2855, and 2930 cm^−1^ are shifted in the case of HAP-Lys compared to their position in pure l-lysine, which indicates the existence of a weak interaction of l-lysine with HAP [36].

### 2.2. PLLA/HAP Scaffolds Physical Properties

**Figure 2:**
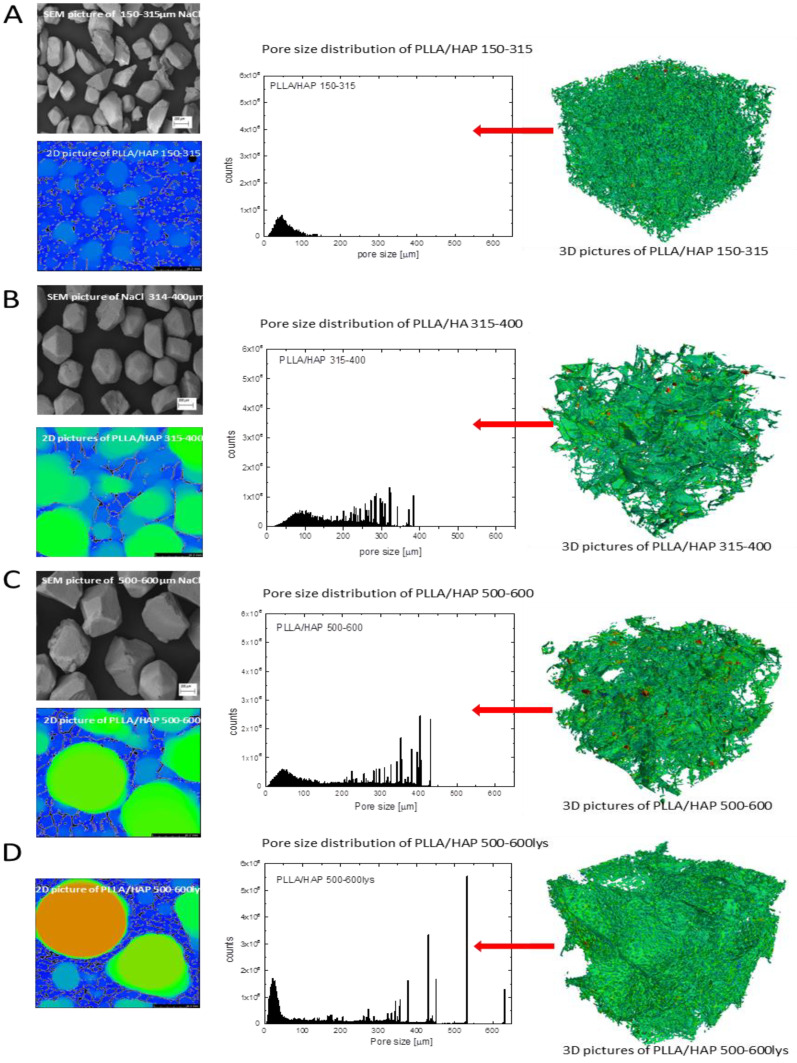
Internal structure of (**A**) poly(l-lactide) (PLLA)/HAP 150–315, (**B**) PLLA/HAP 315–400, (**C**) PLLA/HAP 500–600, (**D**) PLLA/HAP 500–600_lys, sodium chloride SEM (top left), 2D μCT (bottom left), histogram showing pore size distribution (middle) and 3D reconstruction image (right).

Internal, porous structure of the materials was evaluated using µCT as well as SEM and buoyancy techniques. For all the materials porous structure was obtained (Figure 2; Table 1). 

As found earlier by other researchers, the thermally induced phase separation technique usually leads to the formation of pores which are several dozen micrometers in size and have porosity over 80% [20,37,38,39] in the case of polymeric materials. By adding a pore former, usually sodium chloride, which can be removed from the system by leaching, it is possible to obtain pores with greater diameter. In this case, the shape and size of obtained pores are similar to those of used sodium chloride [20]. The NaCl SEM analysis proved differences of the salt size between grains collected from various sieves. No significant differences were found in density and porosity (buoyancy method) for the investigated samples. The density ranged from 0.030 to 0.037g·cm^−3^, while the porosity was in the range of 98.0–98.5% (Table 1). The pore size in the scaffolds rises with that of NaCl used for sample preparation. The µCT analysis proved that for higher sodium chloride grains larger pores were obtained (for PLLA/HAP_150-315 mean pore size = 114.6_± 14.4_; for PLLA/HAP_315–400–226.9± 82.4; for PLLA/HAP_500–600–292.5± 95.8). The results also show that the largest pores might slightly collapse after the salt leaching process because the pores are smaller than expected (Figure 2A–C). The largest pores were obtained for the PLLA/HAP_500–600_lys composite—359.8_±154_ (Figure 2D). These scaffolds were obtained using exactly the same sodium chloride size; however, the sample with l-lysine modified HAP shows greater stiffness so that the pores do not collapse.

The compressive properties are summarized in Table 2. It was found that the compressive modulus of PLLA/HAP_315–400 and PLLA/HAP_500–600 (160–169 kPa) is slightly lower than that of PLLA/HAP_150–300 (185 kPa). 

However, all these values are within the measurement error. The results show that the materials obtained in our research display a much higher stiffness than the corresponding materials with nano-hydroxyapatite [19]. The compressive strength of all the samples is in the range of 20.2–29.6 kPa (at 40% strain) and 147.4–190.0 kPa (at 80% strain). These results are in good correlation with those from our previous study [19].

### 2.3. Biological Evaluation of Osteoblasts Exposed to the PLLA/HAP Scaffolds Viability of hFOB 1.19 Osteoblasts

**Figure 3:**
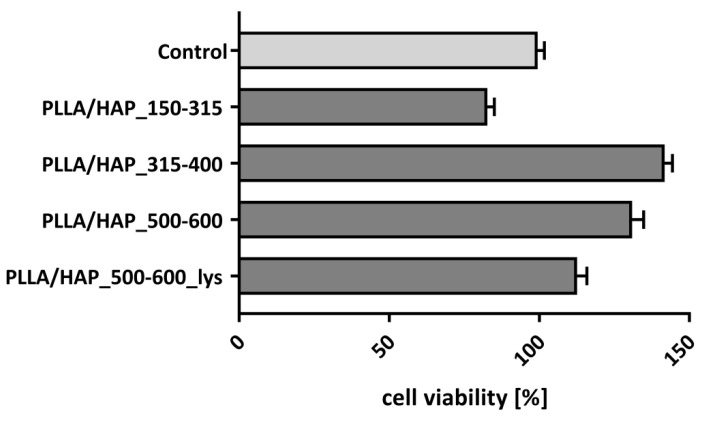
The viability of hFOB 1.19 osteoblasts after 7 days of incubation in the milieu of obtained foam scaffolds. Mean ± SD values obtained in three independent experiments are presented.

The effect of foam scaffolds on the viability/metabolic activity of hFOB 1.19 osteoblasts was determined in a 3-(4,5-dimethylthiazol-2-yl)-2,5-diphenyltetrazolium bromide (MTT) reduction assay, which is based on the ability of the mitochondrial dehydrogenase to convert the water-soluble tetrazolium salt into the insoluble formazan. The colour intensity of dissolved formazan crystals corresponds to the metabolic activity of osteoblasts. Since osteoblasts multiply in a limited manner, we have evaluated the biological activity of foam scaffolds in long term cell cultures, after seven days of incubation. The use of non-adherent cell culture plates enabled us to exclusively evaluate the viability of cells penetrating or adhering to the surface of the biomaterial. Thereby, as shown in Figure 3, the viability of osteoblasts exposed to the PLLA/HAP foam scaffolds differs depending on the pore size and lys-modifications. The hFOB 1.19 viability cultured with PLLA/HAP_150–315, PLLA/HAP_315–400, PLLA/HAP_500–600, and PLLA/HAP_500–600_lys scaffolds reached 87.7 ± 2.4%, 141.8 ± 2.9%, 130.9 ± 2.9%, and 112.6 ± 3.2% respectively, comparing to control cells cultured in the medium without the biomaterials.

Compared to the controls (100% viability), the osteoblasts exposed to foam scaffolds with the smallest pore size (150–315 µm) exhibited the lowest metabolic activity (*p* = 0.03), whereas the incubation with scaffolds containing medium (315–400 µm) or large (500–600 µm) pore size, or scaffolds with large pore size made of material modified with lysine, significantly improved cell viability/metabolic activity: *p* = 0.002, *p* = 0.03, and *p* = 0.04, respectively. The observed cell activity notably demonstrates that these scaffolds do not exhibit cytotoxic effect. This data is in line with the results of Chen-Guang et al., where embryonic osteoblast precursor cells (MC3T3-E1) as well as normal fibroblasts L929 exposed on PLGA scaffolds showed no decrease in viability, even after 72 h [40]. Moreover, their modifications affect metabolic activity or proliferation which was observed in the MTT reduction assay. To address the question whether this phenomenon is a result of the intensification of the adhesion or proliferation of cells remaining in contact with the biomaterials we have performed some more complex experiments described in the following sections of the manuscript. 

#### 2.3.1. hFOB 1.19 Proliferation

**Figure 4:**
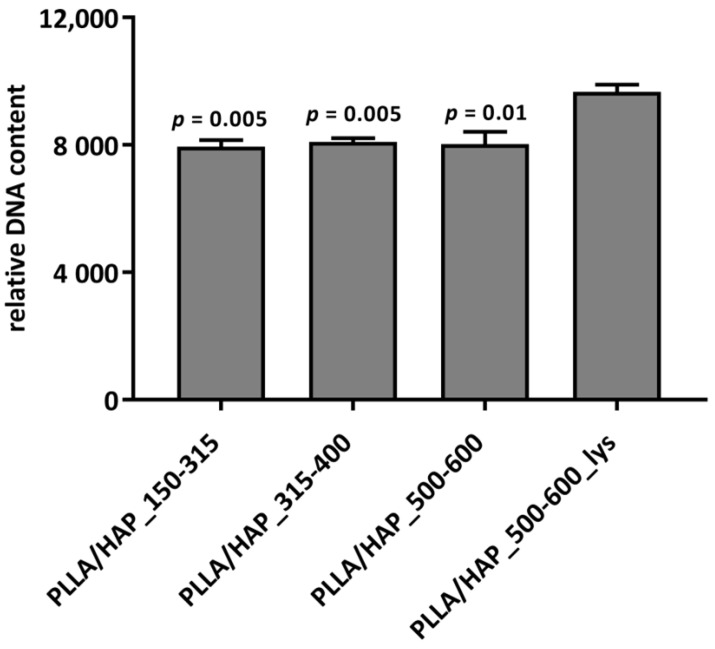
Proliferation of hFOB 1.19 osteoblasts after 7 days of incubation with the PLLA/HAP scaffolds. The results are expressed as mean relative DNA content (RDC) ± SD calculated from three independent experiments. *p* values calculated in comparison to the PLLA/HAP_500–600_lys, one-way ANOVA with post hoc Dunnett’s test.

The number of hFOB 1.19 osteoblasts after seven days of incubation with the PLLA/HAP scaffolds was evaluated quantitatively using the CyQUANT proliferation assay (Figure 4), which is based on the relative fluorescence values determined by the relative DNA content (RDC). We have shown that the PLLA/HAP scaffolds modified with l-lysine stimulated hFOB 1.19 osteoblasts proliferation. The number of cells in cultures incubated with the PLLA/HAP scaffolds with 500–600 µm pores and lysine residues raised significantly (9.6 × 10^3^ ± 94.5 RDC) when compared to the cells exposed to other biomaterials being tested: PLLA/HAP_150–315 (7.9 × 10^3^ ± 214.1 RDC; *p* = 0.005), PLLA/HAP_315–400 (8.0 × 10^3^ ± 122.2 RDC; *p* = 0.05), and PLLA/HAP_500–600 (8.0 × 10^3^ ± 158.8 RDC; *p* = 0.01). Combining these results with the cell activity observed in the previous section, we should conclude that biomaterials with medium and large pores facilitate metabolic activity, whereas the addition of l-lysine promotes cell proliferation. The effect of l-lysine on human osteoblasts in bone regeneration and osteoporosis has already been studied [28,30]. The research of Torricelli et al. indicates that l-lysine may contribute to the activation of cell proliferation by stimulation of the production of platelet derived growth factor, which is highly mitogenic to cells of the osteoblastic origin [28] and may act as an autocrine growth factor.

#### 2.3.2. Cell Attachment and Penetration

**Figure 5:**
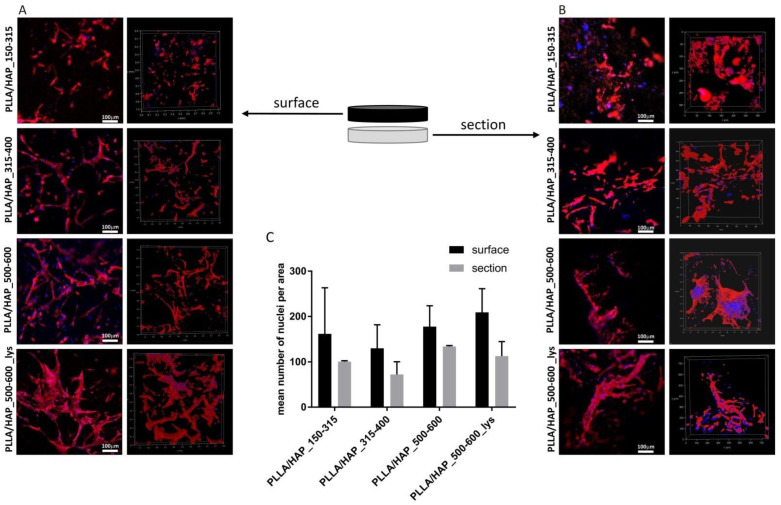
The morphology, adhesion, and penetration of osteoblasts after 7 days of incubation with tested PLLA/HAP foam scaffolds visualized in confocal laser scanning macroscopy (Leica TCS LSI). Cells colonizing the foams on the surface (**A**) or in the section (**B**) were stained with Texas Red-phalloidin (red, F-actin) and 4′,6-diamidino-2-phenylindole (DAPI) (blue, nuclei). Each panel represent 2D (left column) and 3D (right column) pictures of foam scaffolds. To parametrize the obtained results, the nuclei from ten individual image areas were enumerated using ImageJ and presented as the mean number of hFOB 1.19 attached to the surface of (black bars) or penetrating through (grey bars) the foam scaffolds (**C**).

When considering the utility of foam PLLA/HAP composites as candidates for bone regeneration, it is crucial, not only to evaluate their effect on cell viability, but also to verify their potential to support osteoblasts adhesion and penetration into the biomaterial. We have investigated the osteoblasts morphology, adhesion, and penetration after seven days of exposure to different porous scaffolds within their surface and section. We have shown that osteoblasts effectively colonize the scaffolds and penetrate their porous structure; however, with different effectiveness which depends on the pore size and the l-lysine modifications (Figure 5). The PLLA/HAP foams with large pores (500–600 µm) promoted the adhesion of osteoblasts to the surface of the biomaterial more effective than the scaffolds having smaller pores (150–315 µm and 315–400 µm) (Figure 5A). This observation is also confirmed by the enumeration of the cells (nuclei) on the representative biomaterial surface areas (Figure 5C). The number of cells identified on the biomaterial surface (PLLA/HAP_150–315, PLLA/HAP_315–400, PLLA/HAP_500–600, and PLLA/HAP_500–600_lys) is as follows: 161.5 ± 101.8, 130.2 ± 54.3, 177.6 ± 46.1, and 205.6 ± 53.6. Moreover, the modification of the PLLA/HAP scaffolds (500–600 µm) with l-Lysine had the strongest effect on cells’ ability to adhere and elongate on the foam surface. 

Since the cell suspensions were loaded on the top of the materials (in the non-adherent culture vessel) we aimed to evaluate whether the pore size of scaffolds determines the efficiency of cell penetration (Figure 5B). We showed that the cells penetrate the foam scaffolds to a similar extent, irrespective of the pore size of the obtained biomaterial. Moreover, the addition of l-lysine into the foam scaffold with large pores (PLLA/HAP_500–600_lys) did not facilitate the penetration, since the number of cells observed in the section was comparable with the number of cells in the section of PLLA/HAP_500–600 (Figure 5C). However, compared to the number of cells observed on the biomaterial surface, less cells were located deep inside the porous scaffold structure (100.5 ± 2.1, 72.3 ± 28.0, 134.0 ± 2.0, and 112.8 ± 32.8, respectively) (Figure 5C), which may result from inadequate nutrient supply.

#### 2.3.3. The Levels of Calcium Deposited on the PLLA/HAP Scaffolds

**Figure 6:**
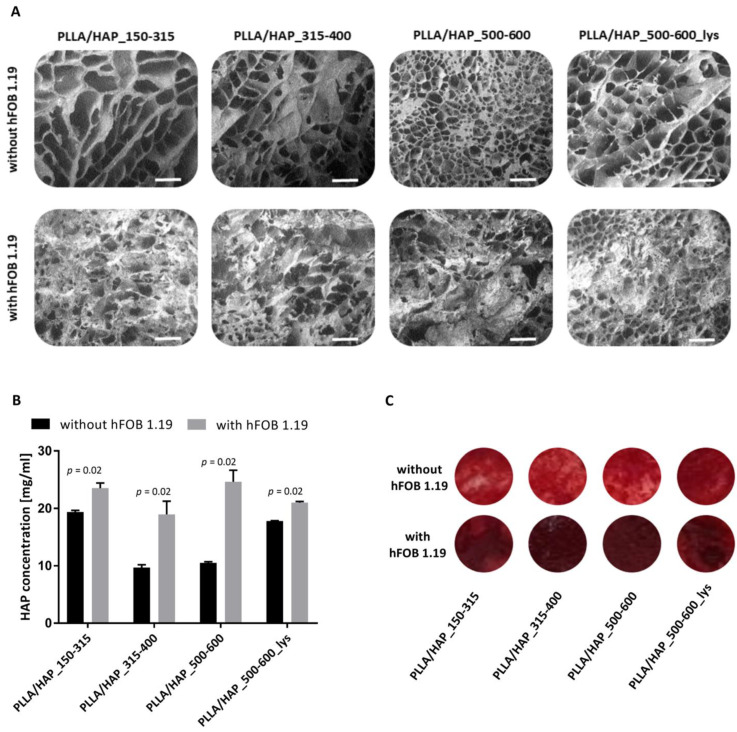
SEM micrographs of the PLLA/HAP scaffolds after 7 days of incubation with hFOB 1.19 osteoblasts or in medium (controls). Scheme 150 m (**A**). Quantification of hFOB 1.19-derived and scaffold-bearing calcium deposits by Alizarin Red S (ARS) staining measured spectrophotometrically at 405 nm. The results are expressed as mean ± SD. *p* values obtained for foam scaffolds with vs. without hFOB 1.19, the Mann–Whitney U test are shown (**B**). The representative distribution of the red pigment created after reaction of ARS with calcium deposits (**C**).

The SEM images showed that hFOB 1.19 osteoblasts cultured in the milieu of foam scaffolds were able to form a cell-derived matrix (Figure 6). Being aware that apatite is one of the components of foam scaffolds, we have evaluated the quantity of calcium deposits present in the crude foam scaffolds and the scaffolds colonized by osteoblasts containing cell debris. Biomineralization of hFOB 1.19 cells cultured on the PLLA/HAP scaffolds was determined quantitatively using Alizarin Red S (ARS) staining method after seven days of culture (Figure 6B). The calcium concentration detected in cultures of osteoblasts exposed to PLLA/HAP_150–315, PLLA/HAP_315–400, PLLA/HAP_500–600, and PLLA/HAP_500–600_lys foams was 23.5 ± 0.7 mg/mL, 19.0 ± 1.9 mg/mL, 24.6 ± 1.6 mg/mL, and 21.0 ± 0.2 mg/mL, respectively, and was significantly higher than that of the control foam scaffolds (without hFOB 1.19): 19.4 ± 0.2 mg/mL, *p* = 0.02; 9.7 ± 0.4 mg/mL, *p* = 0.02; 10.5 ± 0.2 mg/mL, *p* = 0.02 and 17.8 ± 0.1 mg/mL, and *p* = 0.02, respectively. All types of foam scaffolds containing HAP whiskers, regardless the pore size or l-lysine modification induced significant stimulatory effect on the calcium deposits formation in osteoblasts. These observations are consistent with findings of Venugopal et al., who postulate that local partial dissolution of biomaterial and release of calcium and phosphate ions may stimulate the subsequent biomineralization of osteoblasts [41]. This may suggest that extracellular apatite present in the scaffold supports osteoblastic cell adhesion as well as differentiation, which is an important aspect for bone regeneration [42]. A similar effect was observed by Ocampo et al. who, after only seven days of culture of osteoblasts with biomaterial observed biomineralization and detected alkaline phosphatase (ALP), which is an early marker of the differentiation of osteoblasts [43].

Any materials developed for bone tissue engineering and regeneration applications must be biocompatible, which means they cannot have toxic effects. Prior to using biomaterials for in vivo studies, their biocompatibility must be investigated. Based on these results, all of the tested PLLA/HAP scaffolds allow hFOB 1.19 cells to adhere and proliferate with no apparent cytotoxic effect. Moreover, the foam scaffold evaluated in this study can be considered a potential biomaterial to stimulate osteoblast, including hFOB 1.19, to mineralize, which is an important process in bone regenerative medicine.

## 3. Materials and Methods

### 3.1. Materials

The starting calcium phosphate powder (β-TCP, 96%, Product No. 21218) was supplied by Sigma-Aldrich (Poznań, Poland) as a product of Fluka Chemie GmbH, Buchs, Switzerland, and the 30% solution of H_2_O_2_ (Catalog No. BA5193111) was supplied by Avantor Performance Materials Poland S.A., Gliwice, Poland. In this research, we used the poly (l-lactide) Resomer L210s (PLLA) supplied by Evonik, Germany; 1,4-dioxane (CAS:123-91-1) was supplied by Eurochem BGD (Tarnów, Poland) and sodium chloride (CAS: 7647-14-5) for salt leaching experiments was purchased from Stanlab (Lublin, Poland). l-lysine for HAP modification, (CAS: 56-87-1) was purchased from Sigma-Aldrich (Poznań, Poland).

### 3.2. Apatite Whiskers (HAP) Synthesis and Modification with l-Lysine

In our research, multiphasic calcium phosphate whiskers were prepared using the methodology described earlier [31,44]. In brief, 4 g of the β-TCP starting powder were placed in a 250 mL Pyrex glass bottle and 100 mL of a 30% solution of H_2_O_2_ was added. The bottle was capped, shaken for 2 min and heated (undisturbed) in an electric oven at 95 °C for 48 h. The whiskers eventually obtained from the bottle were filtered, washed four times with 500 mL of distilled water and dried overnight at 90 °C.

Modification of HAP with l-lysine was carried out as follows. Initially, 1 g of HAP was dried overnight (at 110 °C), then dispersed in 10 mL of deionized water and sonicated for 5 min. Next, 0.3 g of l-lysine was dissolved in 2 mL of water and added to the HAP suspension. After 24 h of mixing with a magnetic stirrer, the suspension was centrifuged (300 rpm, 5 min) and washed with 10 mL of deionized water. The washing procedure was repeated three times. Finally, the precipitate was dried for the next 24 h at 110 °C. The efficiency of the modification procedure was 92%.

### 3.3. PLLA Foam Scaffolds Preparation

Sodium chloride with appropriate grain size was obtained using a sieve shaker equipped with sieves with 150, 315, 400, 500, and 600 μm eyelets. Three NaCl grain size ranges of 150–315 μm, 315–400 μm, and 500–600 μm were obtained. 

PLLA/HAP composites with wt. ratio of 50/50 were prepared using the procedure described in our previous article [19]. Briefly, PLLA was dissolved in 1,4-dioxane (24 h, magnetic stirrer), after which the apatite ceramic was added to the solution (24 h, magnetic stirrer). Sodium chloride was set in a 24 well plate. Next, the solution of PLLA/HAP in dioxane was added. The 24 well plate was frozen (at −20 °C, 24 h) and freeze dried (−50 °C, 5 Pa, 24 h). The samples were then put in deionized water to solve sodium chloride from the scaffolds. The process of salt leaching took place in a 10l beaker and lasted 24 h; water was changed at least 3 times. The sample list is given in Table 3.

### 3.4. Scanning Electron Microscopy 

To evaluate the morphology and grain size and the morphology & porosity of chloride sodium and PLLA/HAP foams, respectively, a field emission scanning electron microscope (Zeiss, Sigma 500 VP) (Oberkochen, Germany) was used. Before the measurements, the samples were covered with gold (sputter current 40 mA, sputter time 50 s) using a Quorum machine (Quorum International, Laughton, UK) to improve the discharge process.

### 3.5. Compressive Strength

The compressive strength measurements for the foams were performed using a universal INSTRON 5960 (Norwood, MA, USA) equipped with a 1 kN head. The compression speed was set at 2 mm/min. The barrel-shape samples were cut into specimens measuring 4 mm in height and having a diameter of ~15 mm. The samples were tested to obtain the maximum strain of 90%. At least 10 samples of each kind were measured.

### 3.6. Computer Tomography Analysis (CT)

Computer tomography was used to evaluate the pore size distribution of the PLLA/HAP scaffolds obtained using different sodium chloride grain size. The cone beam CT system (GE Phoenix v|tome|x m 300/180—GE Sensing & Inspection Technologies GmbH, Wunstorf, Germany)—was used for the reconstruction of the 3D image of the scaffold. The system is equipped with an X-ray tube with a nanofocus and a diamond target with a maximum accelerating voltage of 180 kV. The tomograph is equipped with a 10-bit detector with dimensions of 40 × 40 cm and a resolution of 2024 × 2024 pixels. During the measurement, the distance between the radiation source and the detector was 808.6 mm, while the distance between the tested object and the radiation source was 10 mm, resulting in a magnification of 80.2× and a resolution of the examination at the level of 2.49 µm. In order to ensure sufficient transmission of radiation through the object, the voltage of the radiation source was set at 50 kV and the intensity at 200 µA. The projection time was 500 ms, and a single projection was obtained by averaging three X-rays.

Three thousand X-ray projections were collected by rotating the sample around its axis. On the basis of the projections obtained in this way, the three-dimensional geometry of the object was reconstructed by the Feldkamp–Kress algorithm (FDK) with filtered backprojection (allowing the reconstruction of three-dimensional geometry from two-dimensional projections, obtained using a cone beam of X-ray) [45]. Data reconstruction (phoenix datos | ×2.7.2) was performed with correction of beam hardening effect, ring artifacts and noise.

Computed tomography data was analysed using the VG Studio MAX 3.3 (Volume Graphics GmbH, Heidelberg, Germany). In the first step, the grayscale data from the tomographic reconstruction was thresholded to determine the volume of the foams. In the next step the Foam/Powder Analysis module of VGSTUDIO MAX was used to create topologically disconnected components that can be visualized and statistically analyzed. The analysis carried out in this way made it possible to determine the size of the pores in the scaffolds.

### 3.7. Porosity Measurements

The porosity of the scaffolds was calculated using the following formula:

(1) $$\Phi_{p}=\left[1-\frac{\rho_{sc}}{\rho_{b}}\right]$$

where *Φ_p_*—porosity, *ρ_sc_*—scaffold density obtained from the buoyancy method using a Hildebrand Electronic Densimeter H-300S, *ρ_b_*—bulk density of the composite scaffold (also measured using H-300S).

### 3.8. Thermogravimetry (TGA)

The thermogravimetric analysis was performed with a TGA/DSC1 Mettler Toledo thermobalance. Samples were heated at a rate of 10 °C/min from 25 °C to 900 °C under 30 mL/min of air flow. For the purpose of data presentation, the TGA curves were exported to OriginPro 64 (v.9.0) (Northampton, MA, USA) as ASCII files. 

### 3.9. Bioefficacy of the PLLA/HAP Foam Scaffolds

**Figure 7:**
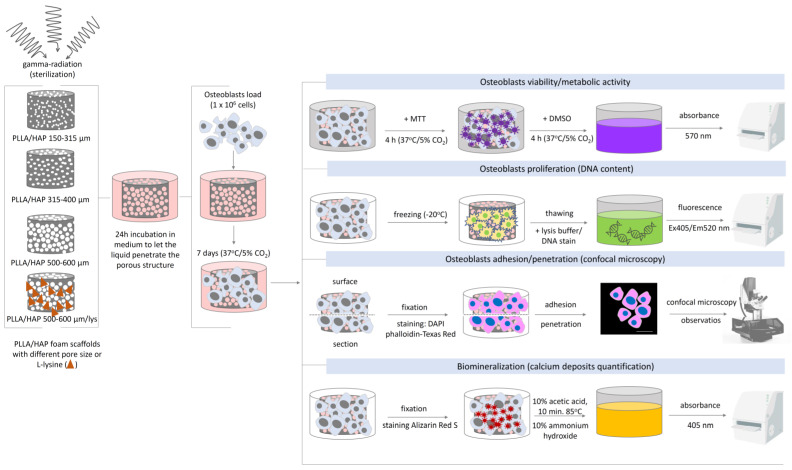
The subsequent stages of the PLLA/HAP foam scaffolds evaluation with regard to their biological interactions with osteoblasts in in vitro studies.

Considering the future utility of the designed foam scaffolds as biomaterials supporting bone regeneration it is necessary to explore their influence on osteoblasts with regard to the metabolic activity/viability, proliferation, and morphology as well as their ability to effectively penetrate the biomaterial and create calcium deposits (Figure 7). 

#### 3.9.1. Sterilization

Prior to the biological evaluation, the PLLA/HAP foam scaffolds were sterilized by gamma-irradiation (as required for biomedical applications) with a dose of 35 kGy gamma rays in a ^60^Co source at the Institute of Applied Radiation Chemistry, Technical University in Lodz (Lodz, Poland). The efficiency of the sterilization process was confirmed for representative foams after incubation in 5 mL of PBS/Tween buffer at room temperature (15 min/shaking) and seeding the liquid (in serial dilutions) on microbiological media intended for bacteria and yeast growth: tryptic soy agar (TSA), and Sabouraud agar incubated for 24 h and 5 days, respectively.

#### 3.9.2. Cell Culture and Propagation

The human fetal osteoblastic cell line hFOB 1.19 (CRL-11372™) obtained from the American Type Culture Collection (ATCC, Manassas, VA, USA) was used in this study. The cells were cultured in a 1:1 mixture of phenol-free Dulbecco’s modified Eagle’s medium and Ham’s 12-F medium (Gibco, Thermo Fisher Scientific, Waltham, MA, USA) supplemented with 2.5 mL of l-Glutamine (100X), 10% heat inactivated fetal bovine serum (FBS) (HyClone Laboratories Inc., Marlborough, MA, USA) and 0.3 mg/mL of geneticin (G418) (Sigma-Aldrich, Saint Louis, MO, USA) at a temperature of 34 °C, in a humidified air atmosphere containing 5% CO_2_. To control the condition of monolayers, the cell cultures were observed daily using an inverted microscope (Motic AE2000, Xiamen, China). The confluent (80–90%) cell monolayers were subcultivated every 2–3 days at a ratio of 1:4 (cell suspension:culture medium) using a 0.25% trypsin (*w*/*v*)-0.5 mM ethylenediaminetetraacetic acid tetrasodium salt (EDTA) solution (Gibco, Thermo Fisher Scientific, Waltham, MA, USA). Prior to each experiment, the cell viability and density were established using a trypan blue exclusion assay and a hemocytometer, respectively.

#### 3.9.3. Direct Contact Cytotoxicity Assay

Under aseptic conditions, each PLLA/HAP scaffold was placed in an individual well of the non-adherent Nunclon Delta Surface 24-well culture plate (Nunc, Thermo Fisher Scientific, Waltham, MA, USA) in four replicates each. During the 24 h incubation in 1 mL of culture medium at 34 °C and 5% CO_2_, the scaffolds absorbed the liquid which filled the porous compartments. Next, the scaffolds were transferred to a new non-adherent 24-well dish and 1 mL of osteoblasts suspension (1 × 10^6^ cells/m) was distributed to each well. Following a 7-day incubation at 34 °C and 5% CO_2_, the cell’s viability was evaluated. First, the culture medium was replaced with 200 μL of fresh culture media and 40 μL of a 3-[4,5-dimethylthiazol-2-yl]-2,5-diphenyltetrazolium bromide solution (MTT) at a concentration of 5 mg/mL (Sigma-Aldrich, Saint Louis, MO, USA) was added to each well and incubated for the next 4 h at 34 °C and 5% CO_2_. The supernatants were replaced with 400 μL of dimethyl sulfoxide (DMSO) (Sigma-Aldrich, Saint Louis, MO, USA) and after 15 min of incubation at room temperature on rotor-shaker, 200 μL of fluid was taken from each well and transferred to a separate 96-well plate. The absorbance was measured at 570 nm using the Multiskan EX reader (Thermo Fisher Scientific, Waltham, MA, USA).

#### 3.9.4. Cell Proliferation Assay

Proliferation of osteoblasts was evaluated after 7 days of incubation with the PLLA/HAP scaffolds using CyQUANT Cell Proliferation Assay (Invitrogen, Thermo Fisher Scientific, Waltham, MA, USA). Following incubation, foam scaffolds containing osteoblasts were carefully washed with PBS to get rid of the non-adherent cells and were frozen at −80 °C. Prior to the DNA quantification, samples were thawed at room temperature and the cells were lysed for 5 min in a buffer containing the CyQuant-GR dye which stains cellular DNA. To evaluate the number of cells and the proliferation rate within the scaffold, serial dilutions of cell suspensions were prepared, and, on their basis, a standard curve was constructed. Fluorescence signals (Ex_405nm/_Em_520nm_) were detected using a SpectraMax^®^ i3x Multi-Mode Microplate Reader (Molecular Devices, San Jose, CA, USA).

#### 3.9.5. Visualization of Cell Adhesion and Penetration 

Foam scaffolds were placed into non-adherent 24-well culture plates (Nunclon Sphera, Nunc, Thermo Fisher Scientific, Waltham, MA, USA) and incubated in the milieu of osteoblasts as described in the previous section. After 7 days of incubation, the foam scaffolds were removed and washed with phosphate buffered saline (PBS), and the cells were fixed with 3.7% paraformaldehyde (Sigma-Aldrich, Saint Louis, MO, USA) for 20 min at room temperature. After the permeabilization (15 min, 0.1% Triton X-100 in PBS), the cells trapped within the scaffolds were stained with fluorescent dyes enabling their visualization by confocal microscopy. To stain the F-actin (cytoskeleton) the preparations were treated with phalloidin conjugated with Texas Red^®^-X (Thermo Fisher Scientific, Waltham, MA, USA) at a final concentration of 1.65 μM (1:40 dilution in PBS) for 40 min. In order to visualize the nuclei, the staining was performed with 2-(4-amidinophenyl)-1H-indole-6-carboxamidine (DAPI) at a final concentration of 1 mg/mL (Thermo Fisher Scientific, Waltham, MA, USA) for 10 min. The remaining dyes were washed 5 times and resuspended in 1 mL of PBS prior to microscopic evaluation. Next, the scaffolds were cut horizontally to create two sections and both the surface, and the section fragments were observed in a confocal macroscope (Leica TCS LSI, Leica Microsystems, Frankfurt, Germany) using a 5×/0.50 LWD objective and imaged with the following fluorescence conditions: Texas Red^®^-X (Ex_596nm_/Em_615nm_) and DAPI (Ex_360nm_/Em_460nm_). The Leica Application Suite X software (LAS X, Leica Microsystems, Frankfurt, Germany) was used for cell imaging. To quantify the mean number of hFOB 1.19 attached to the biomaterial surface, single cells (nuclei) on ten representative areas were enumerated by the ImageJ software v.1.53e (National Institutes of Health, Bethesda, MD, USA). The confocal analysis was performed in the Laboratory of Microscopic Imaging and Specialized Biological Techniques at the Faculty of Biology and Environmental Protection at the University of Lodz.

#### 3.9.6. SEM and Calcium Deposit Quantification

The PLLA/HAP scaffolds were incubated with osteoblasts as indicated previously (Section 3.9.3). Following a 7-day incubation, foam scaffolds were washed with PBS and fixed with a 3.7% paraformaldehyde (Sigma-Aldrich, Saint Louis, MO, USA) for 20 min. The residues of the fixation solution were rinsed with PBS. To visualize the cell matrix within the foam scaffolds in a scanning electron microscope (SEM), after the fixation process (described above), all the samples were dehydrated in ethanol of progressively increasing concentration (10%, 30%, 50%, 70%, 80%, 96%, and 99.6%) for 45 min at each step. The foam scaffolds were examined under a scanning electron microscope PHENOM PRO X SEM (Thermo Fisher Scientific, Waltham, MA, USA) with an accelerating voltage of 15 kV. Next, to detect the osteoblasts-derived and scaffold-containing calcium deposits 1 mL of a 2% solution of 3,4-dihydroxy-9,10-dioxo-9,10-dihydroanthracene-2-sulfonic acid—alizarin red S (ARS) (Sigma-Aldrich, Saint Louis, MO, USA) was added to the foam scaffolds and incubated for 40 min at room temperature with intensive agitation. Then, the excess alizarin red dye was removed, and the scaffolds containing cells were washed 5 times with distilled water. To quantify the calcium deposits, the foam scaffolds were incubated for 30 min in 500 μL of a 10% acetic acid at room temperature with agitation. To completely dissolve and release the red pigment from the foam scaffolds, the samples were heated for 10 min at 85 °C and centrifuged (10 min/400 g). Then the supernatants were transferred to a 96-well microplate and mixed with 200 μL of 10% ammonium hydroxide. The scaffolds incubated for 7 days in a cell culture medium (without osteoblasts) served as a negative (abiotic) control. The optical density was measured at 405 nm using the Multiskan EX reader (Thermo Scientific, Waltham, MA, USA). Values were normalized according to the calibration curve.

#### 3.9.7. Statistical Analysis

Inter-group outcomes were compared for statistical significance using a one-way ANOVA (analysis of variance) with post hoc Dunnett’s test. Intra-group differences were assessed using the non-parametric Mann–Whitney U test. In all the cases, the significance was accepted at *p* < 0.05. All the analyses were performed using GraphPad Prism v.7 (GraphPad Software, San Diego, CA, USA).

## 4. Conclusions

This paper presents a comparison of properties of the PLLA/HAP composite foams obtained using the thermally induced phase separation technique supported by salt leaching process. The samples differed only by the size of sodium chloride used for salt leaching (150–315, 315–400, and 500–600 μm). It was found that all the scaffolds have porosity over 98%, density 0.03–0.037 g × cm^−3^ and pore size distribution corresponding to the grains size of sodium chloride used for the preparation of scaffolds. All the materials have comparable compressive parameters irrespective of the pore size.

Compared to the scaffolds with smaller pores, (150–315 µm and 315–400 µm), the PLLA/HAP foams with large pores (500–600 µm) promote a more effective adhesion of osteoblasts to the surface of the biomaterial. Osteoblastic cells penetrate the foam scaffolds to a similar extent, irrespective of the pore size of the obtained biomaterial. Moreover, the addition of l-lysine modified HAP into the foam scaffold with large pores (PLLA/HAP_500–600_lys) did not facilitate the penetration, since the number of cells observed in the section was comparable with the number of cells in the section of PLLA/HAP_500–600. In the research, no benefits were observed for PLLA foams with l-lysine modified HAP.

## Figures and Tables

**Table 1:** Density and porosity of the scaffolds (mean value _± standard deviation_).

Sample	Density *ρ_sc_* (g·cm^−3^)	Porosity *Φ_p_* (%)
PLLA/HAP_150–315	0.037_±0.005_	98.0_±0.1_
PLLA/HAP_315–400	0.030_±0.002_	98.4_±0.1_
PLLA/HAP_500–600	0.032_±0.005_	98.2_±0.3_
PLLA/HAP_500–600_lys	0.036_±0.004_	98.5_±0.2_

**Table 2:** Compressive properties of the scaffolds (mean value _± standard deviation_).

Sample	Compressive Modulus (kPa)	Compressive Stress (40%strain) (kPa)	Compressive Stress (80%strain) (kPa)
PLLA/HAP_150–315	185_±23_	24.3_±2.5_	190_±17.9_
PLLA/HAP_315–400	160_±11_	20.2_±1.2_	147.4_±7.8_
PLLA/HAP_500–600	169_±23_	29.6_±3.0_	169.3_±23.9_
PLLA/HAP_500–600_lys	250_±25_	26.5_±3.9_	173.8_±20.3_

**Table 3:** List of the samples investigated in the study.

Sample Designation	Apatite Content (wt.%)	Size of Sodium Chloride Used for the Salt Leaching Procedure (μm)
PLLA/HAP 150–315	50	150–315
PLLA/HAP 315–400	50	315–400
PLLA/HAP 500–600	50	500–600
PLLA/HAP 500–600_lys	50	500–600

## Data Availability

The data presented in this study are available within the article. Other data that support the findings of this study are available upon request from the corresponding authors.
